# VIP Gene Deletion in Mice Causes Cardiomyopathy Associated with Upregulation of Heart Failure Genes

**DOI:** 10.1371/journal.pone.0061449

**Published:** 2013-05-20

**Authors:** Anthony M. Szema, Sayyed A. Hamidi, S. David Smith, Helene Benveniste

**Affiliations:** 1 Veterans Affairs Medical Center, Northport, New York, United States of America; 2 Department of Medicine, Stony Brook University, Stony Brook, New York, United States of America; 3 Brookhaven National Laboratory, Upton, New York, United States of America; 4 Departments of Anesthesiology and Radiology, Stony Brook University, Stony Brook, New York, United States of America; University of Otago, New Zealand

## Abstract

**Rationale:**

Vasoactive Intestinal Peptide (VIP), a pulmonary vasodilator and inhibitor of vascular smooth muscle proliferation, is absent in pulmonary arteries of patients with idiopathic pulmonary arterial hypertension (PAH). We previously determined that targeted deletion of the VIP gene in mice leads to PAH with pulmonary vascular remodeling and right ventricular (RV) dilatation. Whether the left ventricle is also affected by VIP gene deletion is unknown. In the current study, we examined if VIP knockout mice (VIP^−/−^) develop both right (RV) and left ventricular (LV) cardiomyopathy, manifested by LV dilatation and systolic dysfunction, as well as overexpression of genes conducive to heart failure.

**Methods:**

We examined VIP^−/−^and wild type (WT) mice using Magnetic Resonance Imaging (MRI) for evidence of cardiomyopathy associated with biventricular dilation and wall thickness changes. Lung tissue from VIP^−/−^ and WT mice was subjected to whole-genome gene microarray analysis.

**Results:**

Lungs from VIP^−/−^ mice showed overexpression of cardiomyopathy genes: Myh1 was upregulated 224 times over WT, and Mylpf was increased 72 fold. Tnnt3 was increased 105 times and tnnc2 181 fold. Hearts were dilated in VIP^−/−^ mice, with thinning of LV wall and increase in RV and LV chamber size, though RV enlargement varied. Weights of VIP^−/−^ mice were consistently lower.

**Conclusions:**

Critically-important heart failure-related genes are upregulated in VIP^−/−^ mice associated with the spontaneous cardiomyopathy phenotype, involving both left and right ventricles, suggesting that loss of the VIP gene orchestrates a panoply of pathogenic genes which are detrimental to both left and right cardiac homeostasis.

## Introduction

Idiopathic (primary) pulmonary arterial hypertension (PAH) is a relatively uncommon [1] but highly fatal disease [2] characterized by progressive PAH and increased thickening of smaller pulmonary arteries and arterioles, culminating in right ventricular (RV) failure [3]. Considerable advances have been made in recent years in our knowledge of the pathophysiology, pathology, and genetic basis of PAH disease [4] and its treatment is now more successful [5]. However, the underlying pathogenetic mechanisms of the disease, particularly the interactions among multiple predisposing genes, and the influence of selected environmental factors are still poorly understood.

Previously, we reported that mice with targeted deletion of the VIP gene (VIP^−/−^) have moderate PAH that occurs spontaneously – that is, without initiators such as hypoxia or ingestion of agents such as *crotalaria spectabilis* seeds known to induce PAH [6]. We also reported that the medial wall of pulmonary arteries (45 to 50 µm diameter), was significantly thicker and the lumen was significantly narrower in VIP^−/−^ mice compared to control WT mice [6]. In fact, the most striking abnormality observed in VIP^−/−^ was that numerous pulmonary vessels were so severely narrowed they appeared almost totally occluded. Further, clusters of inflammatory cells, predominantly mononuclear infiltrates, were observed around smaller pulmonary vessels and airways [6]. Using invasive post-mortem techniques, we further documented that the (RV septum/LV+septum) weight ratio, in male VIP^−/−^ mice was significantly higher than in male control WT mice – suggestive of relative RV hypertrophy.

The likelihood of left ventricular and right ventricular impairment contributing to the origin of pulmonary arterial hypertension in the VIP^−/−^ mice rather than representing secondary sequelae is highlighted by the lack of arterial hypoxemia or systemic hypertension in these mice [6]. These mice also have features of clinical asthma, with peribronchiolar airway inflammation, airway hyper-responsiveness to inhaled methacholine, and upregulation of pro-inflammatory cytokines [7]. Such features may potentially influence right ventricular function [8].

In the present study, we use non-invasive *in vivo* high resolution magnetic resonance imaging (microMRI) to first test the hypothesis that both the LV and RV are affected in VIP^−/−^ mice, leading to a biventricular dilated cardiomyopathy secondary to pulmonary hypertension. Second, gene microarray analyses of lung tissue were performed to characterize potential differences in the expression of cardiomyopathy genes between VIP^−/−^ and normal mice. Specifically, we hypothesized that VIP^−/−^ mice would overexpress cardiomyopathy genes in comparison to WT mice.

While the effects of VIP on smooth muscle proliferation are well-known [9]–[14], the effect on striated muscle has not been well-studied. Our study is the first to test the effect of loss of the VIP gene on striated left and right ventricular muscle and function, supporting the role for VIP as potential therapeutic option in cardiomyopathy.

## Materials and Methods

### Animals

VIP^−/−^ mice, backcrossed to C57BL/6 mice, were prepared locally as described earlier [7] and genotyped to confirm the absence of the VIP gene. We mated homozygous (VIP^−/−^) males with homozygous (VIP^−/−^) females or, if necessary, with heterozygous (VIP^−/+^) females. For genotyping, we extracted DNA from 1-cm-long tail snips using a DNA isolation kit (Qiagen Inc., Valencia, Calif). DNA (100 ng) was subjected to polymerase chain reaction using primers to detect both VIP and the neomycin cassette. Control, wild-type (WT) C57BL/6mice were from Taconic Labs (Germantown, NY). We examined animals ranging in age from 9 to 52 weeks. The entire study was approved by the institutional animal review committees at Brookhaven National Laboratory and VAMC Northport.

### MRI

#### Animals and preparation for MR imaging

A total of fourteen male mice were used for the studies (**Table 1**); Group 1  =  Control; C57BL6/J male mice (n = 7) and Group 2  =  (VIP^−/−^) male mice (n = 7). The mean age of the control mice and VIP^−/−^ mice were 10.6 months (10.6±1.7 months) and 11.7 (11.7±0.2 months), respectively. For MR imaging all animals were initially anesthetized with an intra-peritoneal injection of a barbiturate (Nembutal®, 20 mg/kg) in addition to an antisialagogue glycopyrrolate (0.01–0.02 mg/kg) used to reduced airway secretions.1–2% isoflurane vapor in a 1∶1 air: oxygen mixture was delivered via a nose cone to the spontaneously breathing animal for anesthesia maintenance during the scan. The electrocardiogram (ECG), respiratory rate, heart rate and body temperature was continuously monitored (SA Instruments). All MRI imaging were initialized when the body temperature was stabilized at 36.6–37°C.

**Table 1:** Body weight and heart rate recorded from mice during MR imaging.

	Group 1; Control (n = 7)	Group 2; VIP^−/−^ (n = 6)	P value
Body weight (g)	41.2±9.6	32.3±4.3*	0.038
Heart rate (bpm)	529±37	549±24	0.360

The mean body weight in VIP^−/−^ mice was reduced compared to WT mice.

#### *In vivo* MR imaging

**Figure 1:**
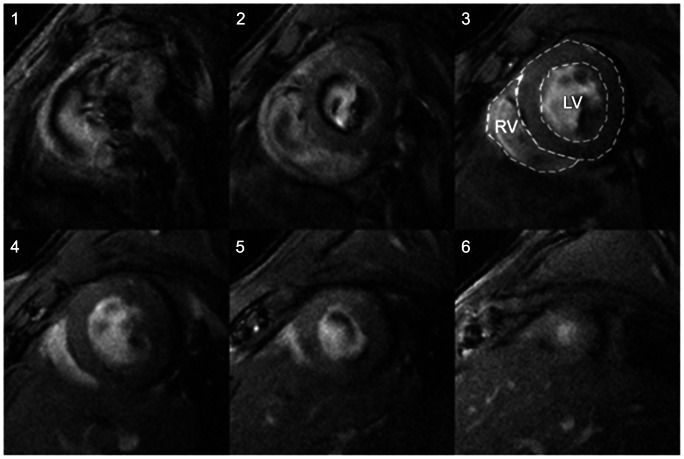
End-diastolic multi-slice MR images acquired in the coronal plane orientation (short-axis view) through a normal mouse heart. We used slice 3, 4 and 5 for data analysis of right and left ventricle volumes. Dotted lines demonstrate as an example the areas for RV and LV volume calculation in one of the mid-ventricular slices.

MR images were acquired on a 9.4T horizontal bore Bruker magnet using a 12-cm volume radio-frequency coil for signal excitation with a 2-cm surface coil for acquisition. A new animal cradle system designed to reduce vibration, provide better animal positioning and isolation of the volume coil during MR imaging was used for the studies. The mouse was positioned supine in the cradle with the 2-cm surface coil position above the heart. The correct image plane localization was determined from scout images acquired in the vertical and horizontal left-ventricle (LV) axis. Short axis bright blood views of the heart were obtained with an ECG gated multi-slice 2D-FLASH gradient echo sequence: TR  = 7.5 ms, TE  = 2.9 ms, Flip angle = 10°, in-plane-resolution: 0.133×0.133 mm^2^, slice thickness: 1mm; interslice gap  = 0.5 mm. The heart rate and consequent R–R interval typically allowed for acquisition of 12 cine frames within each cardiac cycle with the given temporal resolution of 7.2 ms. Typically 5–6 short-axis slices covered the total heart; from the level of the heart valves to the apex (**Figure 1**) however, only three short axis slices displaying right and left ventricle clearly during the cardiac cycle was used for analysis.

Image analysis was performed using the freely available cardiac analysis software [15]. For all studies, end-systole was referred to as the frame with minimal ventricular cavity volume. Right ventricular (RV) and LV volume measurements were obtained by outlining the RV and LV areas in each of the three consecutive short-axis slices which were selected for the data analysis (cf. **Figure 1**). The RV and LV cavities were outlined first using the semi-automated algorithm in the software Segment followed by a manual correction. The post-hoc manual correction was necessary especially on one or two cine frames acquired near end-diastole and mid-diastole where ‘black-blood’ artifacts were present that arise from inflowing blood when using the FLASH gradient-echo sequence. RV and LV volume estimates were subsequently calculated by multiplying the single-slice areas with the slice-thickness and adjusting for the 0.5-mm inter-slice gap. The average ES LV wall thickness was also measured in the three short-axis slices.

### Gene microarray analysis

Agilent whole-genome microarray analysis: Lungs from five male VIP^−/−^ and five male WT mice, 20–24-weeks old, were removed from freshly euthanized animals, immersed in RNAlaterTM (10 mL per 1 mg of tissue; Ambion, Austin, TX, USA), fresh-frozen in liquid nitrogen and shipped overnight on dry ice to Superarray Biosciences (Fredrick, MD, USA). Microarray data were collected at Superarray Biosciences, using the Whole Mouse Genome Oligo Microarray Kit with SurePrint technology (4*6*44K slide format; Agilent Technologies, Palo Alto, CA, USA).

### Statistical analysis

All data are presented as mean ± SD. Statistical analyses were performed using XLSTAT Version 2011 (Addinsoft, NY, USA) with p-value<0.05 described as significantly different. Differences in body weight and heart rate between the control and VIP^−/−^ mice were compared using a two-sided t-test for independent groups. Differences between cardiac morphometric parameters and EF between the two groups were compared using a two-sided t-test and statistical significance was set at p<0.007 after Bonferroni adjustment.

**Table 5:** Force Generation & Propagation.

Gene symbole	Gene name	KO/WT (fold change) *
MYl1	Myosine light chain 1	21
Mylk	Myosine light	11
Myl3	Myosine light chain 3	30
Mylpf	Myosine light polypepdase kinase	72
MYlk2	Myosine light chain kinase 2	9
Myh1	Myosine heavy chain 1	224
Myh2	Myosine heavy chain 2	10
Myh4	Myosine heavy chain4	29
Myh8	Myosine light chain 8	44
TPM1	Tropomyosin 1	3
ACTC1	Cardiac actin	2
tnnt3	Troponin T3, skeletal, fast	105
tnni2	Troponin, skeletal, fast 2	83
tnnc2	Troponin C2, fast	181
ttn	Titin	10
***Intermediate filaments and dystrophin-associated glycoprotein mutations***		
sgca	sarcoglycan	3.5
cav3	caveolin	3.6
snat1	syntrophin	0.8
dtna	dystrobrevin	0.7
***Mutations in intercalated and Z-disc proteins***		
csrp3	cysteine and glycine-rich protein 3	3
tcap	telethonin	1.7
***Lamin A/C mutations.***		
emd	emerin	0.8
* ***P*** **<0.05**		

## Results

### MRI cardiac phenotyping of VIP^−/−^ mice versus controls

The body weights of the VIP^−/−^ mice were significantly lower than control mice (**Table 1**). However, average heart rates measured during MRI between the control and VIP^−/−^ mice were within similar range (**Table 1**).

**Figure 2:**
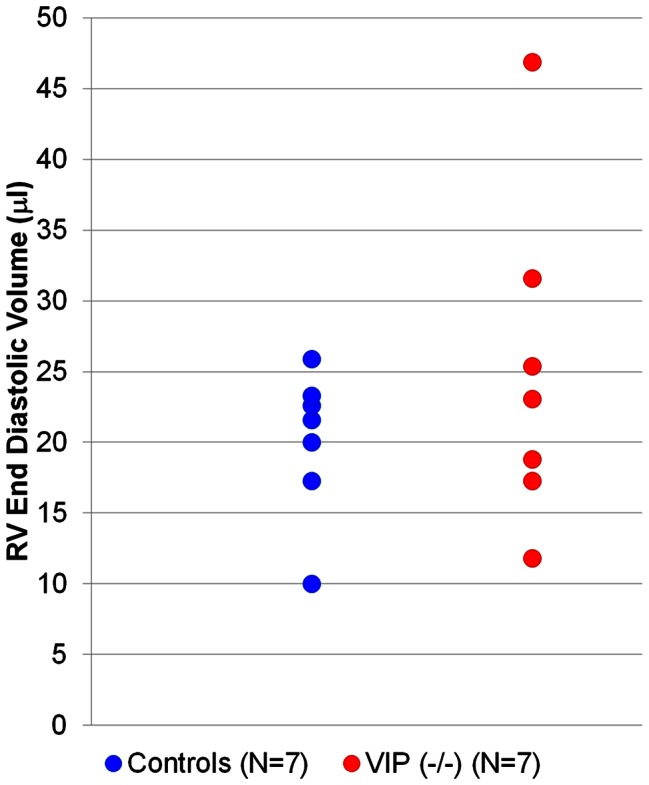
Scatter gram of RV ED volumes measured in the short-axis MRIs of individual control mice (blue) and VIP^−/−^ mice (red circles) demonstrating that the RV phenotype varied in the VIP^−/−^ mice.

**Figure 3:**
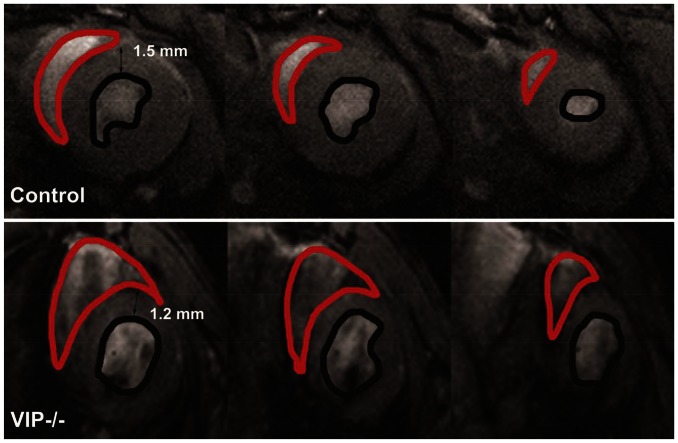
End-diastolic short-axis MR images through a control mouse heart (top panel) and a VIP^−/−^ mouse (bottom panel). The RV and LV endocardium has been outlined in red and black, respectively. It is clear that the VIP^−/−^RV is dilated compared to the control mouse. Quantitative measures also show that the LV wall of the VIP^−/−^mouse is thinner than that of the control mouse.

**Table 2** shows LV and RV dynamic volume data for control and VIP^−/−^ mice and demonstrates that LV wall thickness was significantly lower in the VIP^−/−^ mice compared to controls (p-value <0.01) suggestive of cardiomyopathy. Although the average RV ESV and EDV of the VIP^−/−^ mice were no different from controls, two of the VIP^−/−^ mice exhibited moderate to severe RV pathology; suggesting that the cardiac phenotype is variable. **Figure 2** shows the distribution of RV EDV values in individual VIP^−/−^ mice compared to controls, and **Figure 3** shows short-axis cardiac MRIs from one of the VIP^−/−^ mice with the most severe RV dilated cardiomyopathy compared to a normal control mice.

**Table 2:** LV and RV data for the control and VIP^−/−^ mice.

Parameter	Control (n = 7)	VIP^−/−^ (N = 7)	P value
LV ESV (µl)	13.0±4.1	13.7±3.9	0.75
LV EDV (µl)	41.2±7.2	40.0±6.9	0.77
LV average ES wall thickness (mm)	1.6±0.1	1.1±0.3	0.005
LV EF (%)	68.9±4.7	65.7±6.7	0.33
RV ESV (µl)	8.4±3.3	13.9±13.2	0.31
RV EDV (µl)	20.1±5.2	25.0±11.5	0.33
RV EF (%)	59.0±11	51.0±19.6	0.38

**Table 6:** Energy Production & Regulation.

Gene symbol	Gene name	KO/WT (fold change) *
PRKAG2	AMP-activated protein kinse	0.8
* ***P*** **<0.05**		

**Table 7:** Ca^2+^ Cycling.

Gene symbol	Gene name	KO/WT (fold change) *
Fkbp1b	Castabin2	0.7
* ***P*** **<0.05**		

### Gene microarray analysis

Data obtained from the whole-genome microarray analysis was filtered with respect to the genes involved in pulmonary vascular remodeling and cardiomyopathy. All the changes in gene expression obtained by microarray analysis using the lungs of VIP^−/−^ and WT mice (**Table 3**). Lungs from VIP^−/−^mice showed significant alterations in the expression of several genes pertinent to pulmonary vascular tone, vascular remodeling, and lung inflammation. In addition, we found alteration in genes related to hypertrophic/dilated cardiomyopathy (**Table 4**).

**Table 3:** Gene expression alterations in VIP^−/−^ mice compared to WT mice.

*Gene*	*Symbol*	*Microarray (KO)*	
		*Change*	*VIP KO/WT*
*Vasoconstrictor, pro-remodeling genes:*			
myosin, heavy polypeptide 1	Myh1	↑	225.00
myosin, heavy polypeptide 8	Myh 8	↑	44.00
myosin, light polypeptide 3	Myl3	↑	30.00
procollagen, type V, alpha 1	Col5a1	↑	1.20
Rho, GDP dissociation inhibitor (GDI) beta	Arhgdib	↑	1.48
platelet derived growth factor receptor b	pdgfrb	↑	1.20
*Vasodilator, anti-remodeling genes:*			
vasoactive intestinal polypeptide	Vip	↓	0.04
adrenergic receptor, beta 2	Adrb2	↓	0.37
apolipoprotein E	Apoe	↓	0.76
bone morphogenetic protein 2	Bmp2	↓	0.86
nitric oxide synthase 3, endothelial cell	Nos3	↓	0.60
GTP cyclohydrolase 1	Gch1	↓	0.77
prostaglandin I2 (prostacyclin) synthase	Ptgis	↓	0.76
vascular endothelial growth factor C	Vegfc	↓	0.58
*Inflammatory genes:*			
nuclear Factor of Activated T cell	NFATc2	↑	1.40
chemokine (c-c motif) receptor 6	Ccr6	↑	2.52
mast cell protease 8	Mcpt8	↑	7.00
tumor necrosis factor	Tnf	↑	1.90

**Table 4:** Genes alterations Related to Hypertrophic/Dilated Cardiomyopathy in VIP^−/−^ mice compared to WT mice.

Gene symbole	Gene name	KO/WT (fold change)	P value
casq1	calsequestrin 1	19	0.004
casq2	calsequestrin 2	2	0.050
ttn	Titin (connectin)	10	0.004
cd59a	cd59a	3.4	0.002
cd59b	cd59b	1.5	0.007
lum	lumican	3.3	0.010
TPM1	alpha tropomyosine	2.9	0.008
des	desmin	2.9	0.024
dcn	Deconin	1.7	0.040
ACTC1	Cardiac actin	1.6	0.070

**Table 8:** Transcriptional Regulators.

Gene symbol	Gene name	KO/WT (fold change) *
nfatc3	Neuclear Factor of Activated T cell	1.14
Myoz2	Calsacin-1	78
* ***P*** **<0.05**		

Families of deleterious genes conducive to vascular remodeling and cardiac hypertrophy are upregulated in VIP^−/−^ mice compared to control mice. In particular myosin heavy peptide 1 is increased 225 fold.

Cardiomyopathy genes may be organized based on function: 1) force generation and propagation, 2) energy production and regulation, 3) calcium cycling, and 4) transcriptional regulation [16].

## Discussion

VIP^−/−^ mice have a cardiac phenotype of cardiomyopathy of the LV and RV with pulmonary hypertension in a setting of heart failure gene upregulation. Our earlier finding of right ventricular dilation and primary pulmonary hypertension is further supported by the current data, not in isolation, so that it is possible that left ventricular failure may also contribute to right heart failure. The significance of bilateral cardiomyopathy in VIP^−/−^ mice is that this links the VIP gene to critical whole heart myosin gene overexpression, tied to abnormal function in its absence, and supports the concept of VIP therapy for cardiac disease.

A larger variation in right ventricle dimensions compared to that of the left ventricle may result from the heterogeneity in expression of the asthma phenotype that is seen among the homozygous VIP knockout mice [7]. Asthma-like features and pulmonary hypertension co-exist in this strain [7]. Our earlier work indicating normal left ventricular pressures and high pulmonary artery pressures in the absence of hypoxemia support the primary cause related to lack of VIP.

The phenotype which is visually evident particularly in 3-D (online supplement) shows that the RV is clearly dilated with a reverse D-shaped chamber and poor contractile properties. There is turbulence within the RV chamber since very dark areas demonstrate contrast in a non-homogeneous fashion. The LV is thinned, with poor “squeeze” suggesting inability to contract functionally. Upregulation of selected cardiomyopathy genes correlate with 9.4T microMRI data.

Each of the key mechanisms in cardiomyopathy is affected: 1) force generation and propagation, 2) energy production and regulation, 3) calcium cycling, and 4) transcriptional regulation [16].

Although some cases of human idiopathic pulmonary arterial hypertension (IPAH) are due to autosomal dominant mutations in the BMPR2 gene, most patients with IPAH do not have this mutation [17]. Other investigators have correlated a deficiency of serum and lung tissue levels of VIP with IPAH though mutations in the VIP gene have not been identified in these patients [18].

VIP knockout humans have not yet been identified. Nevertheless, overexpression of cardiomyopathy genes in VIP^−/−^ mice supports the concept of VIP's potential role in therapy for heart failure.

The VIP^−/−^ model may help elucidate underlying pathogenetic mechanisms of the disease, particularly the interactions among multiple predisposing genes. While the influence of selected environmental factors are still poorly understood, use of this model concurrent with hypoxia chambers or monocrotolaria may allow us to dissect the complex interplay between genes and the environment in pulmonary arterial hypertension.

Of all the left ventricular cardiac parameters measured, only left ventricular wall thickness was different. In patients, the relationship between LV and RV function may also relate to PAH. In a cohort of IPAH patients after therapy with a vasodilator, there was reduction of RV systolic pressure and LV preload with increases in stroke volume, suggesting that RV load reduction might impact LV diastolic function in PAH via altering interventricular septal interactions [19].

### 1) Force Generation and Propagation (Table 5)

#### Sarcomere protein mutations

Gene mutations in humans for the genes encoding protein components of the sarcomere have been associated with both hypertrophic and dilated cardiomyopathy. Specific molecular control of functions – remodeling, histopathology, hemodynamic profiles, and biophysical consequences – lead to the final common pathway of heart failure. Hundreds of dominant mutations in genes encoding β-cardiac myosin heavy chain (MYH7), cardiac myosin-binding protein-C (MYBPC3), cardiac troponin T (TNNT2), cardiac troponin I (TNNI3), essential myosin light chain (MYL3), regulatory myosin light chain (MYL2), α-tropomyosin (TPM1), cardiac actin (ACTC), and titin (TTN) have been reported to cause HCM [20]. Comprehensive sequencing of sarcomere protein genes in diverse patient populations indicates that MYBPC3 and MYH7 mutations are most frequent [4], [5]. Sarcomere gene mutations that cause HCM produce a shared histopathology with enlarged myocytes that are disorganized and die prematurely, which results in increased cardiac fibrosis. The severity and pattern of ventricular hypertrophy, age at onset of clinical manifestations, and progression to heart failure are in part dependent on the precise sarcomere protein gene mutation. For example, TNNT2 mutations in patients are generally associated with a high incidence of sudden death despite only mild left ventricular hypertrophy [21], [22]. While only a small subset (10–15%) of HCM patients develop heart failure, this end-stage phenotype has a markedly poor prognosis and often necessitates cardiac transplantation. Accelerated clinical deterioration has been observed with MYH7 Arg719Trp, TNNT2 Lys273Glu, TNNI3 Lys183del, and TPM1 Glu180Val mutations [23]–[26].

While specific myosin light chain and troponin genes differ between mice and man, the phenotype of dilated cardiomyopathy in a setting of overexpression of these sarcomere protein genes suggests that overexpression may be a compensatory mechanism.

#### Intermediate filaments and dystrophin-associated glycoprotein mutations

Intermediate filaments function as cytoskeletal proteins linking the Z-disc to the sarcolemma. Desmin is a type III intermediate filament protein, which, when mutated, causes skeletal and cardiac muscle disease. The hearts of mice deficient in desmin [27] are more susceptible to mechanical stress, which is consistent with the function of intermediate proteins in force transmission. Through dystrophin and actin interactions, the dystrophin-associated glycoprotein complex (composed of α- and β-dystroglycans, α-, β-, γ- and δ-sarcoglycans, caveolin-3, syntrophin, and dystrobrevin) provides stability to the sarcomere and transmits force to the extracellular matrix.

Upregulated expression of sarcoglycan and caveolin genes in VIP KO mice supports the concept of increased force to the extracellular matrix and ventricular hypertrophy, presumably from protein products of these genes having a functional role in force transmission.

#### Lamin A/C mutations

The inner nuclear-membrane protein complex contains lamin. Dominant lamin A/C mutations exhibit cardiac-restricted phenotype with fibrofatty degeneration of the myocardium and conducting cells, although subclinical involvement of skeletal muscles and contractures are sometimes apparent.

Lamin gene over-expression in VIP KO mice would presumably protect from fibrofatty degeneration of the myocardium, which is not seen histologically. Rather, increased RV/LV+septum weight and heart size divided by mouse weight is present.

### 2) Energy Production and Regulation (Table 6)

Nuclear-encoded metabolic gene mutations affecting key regulators of cardiac metabolism are emerging as recognized causes of hypertrophic cardiac remodeling and heart failure. Mutations in genes encoding the γ2 subunit of AMP-activated protein kinase (PRKAG2), α-galactosidase A (GLA), and lysosome-associated membrane protein-2 (LAMP2) can cause profound myocardial hypertrophy in association with electrophysiologic defects [28]. AMP-activated protein kinase functions as a metabolic-stress sensor in all cells. This heterotrimeric enzyme complex becomes activated during energy-deficiency states (low ATP, high ADP) and modulates (by phosphorylation) a large number of proteins involved in cell metabolism and energy [29].

Glucagon and VIP increase intracellular cyclic amp and may be useful in cases of anaphyalxis not responsive to epinephrine (patient taking beta blockers) or calcium channel blocker overdose. Lack of VIP in VIP^−/−^mice leading to upregulation PRKAG2, a subunit of the AMP-activated protein kinase would appear to be an overwhelming compensatory response.

### 3) Ca^2+^ Cycling (Table 7)

Considerable evidence indicates the presence of abnormalities in myocyte calcium homeostasis to be a prevalent and important mechanism for heart failure. Protein and RNA levels of key calcium modulators are altered in acquired and inherited forms of heart failure, and human mutations in molecules directly involved in calcium cycling have been found in several cardiomyopathies.

Calcium enters the myocyte through voltage-gated L-type Ca2+ channels; this triggers release of calcium from the sarcoplasmic reticulum (SR) via the RyR2. Emerging data define FK506-binding protein (FKBP12.6; calstabin2) as a critical stabilizer of RyR2 function [30], preventing aberrant calcium release during the relaxation phase of the cardiac cycle.

If mouse FKBP serves the same function as human FKB as a critical stabilizer of RyR2 function, then it would follow that overexpression of FKBP in mice could lead to aberrant, excess calcium release during the relaxation/diastolic phase of the cardiac cycle, enhances the opportunity to generate hypertrophy.

### 4) Transcriptional Regulators (Table 8)

Mechanisms that activate or repress cardiac gene transcription may induce key molecules that directly or indirectly lead to cardiac remodeling. While human mutations in these genes have not been identified, these molecules are excellent candidates for triggering cell responses to structural protein gene mutations.

Hypertrophic remodeling is associated with reexpression of cardiac fetal genes. Molecules that activate this program may also regulate genes that directly cause hypertrophy. Activation of calcineurin (Ca^2+^/calmodulin-dependent serine/threonine phosphatase) results in dephosph-orylation and nuclear translocation of nuclear factor of activated T cells 3 (NFAT3), which in association with the zinc finger transcription factor GATA4, induces cardiac fetal gene expression. Transgenic mice that express activated calcineurin or NFAT3 in the heart develop profound hypertrophy and progressive decompensation to heart failure [31], responses that were prevented by pharmacologic inhibition of calcineurin. Although these data implicated NFAT signaling in hypertrophic heart failure, pharmacologic inhibition of this pathway fails to prevent hypertrophy caused by sarcomere gene mutations in mice and even accelerates disease progression to heart failure [32]. Mice lacking calsarcin-1, which is localized with calcineurin to the Z-disc, showed an increase in Z-disc width, marked activation of the fetal gene program, and exaggerated hypertrophy in response to calcineurin activation or mechanical stress, which suggests that calsarcin-1 plays a critical role in linking mechanical stretch sensor machinery to the calcineurin-dependent hypertrophic pathway [31].

NFAT expression inducing cardiac fetal gene expression would explain the profound RV hypertrophy and risk for heart failure in VIP^−/−^ mice. Our preliminary data also indicate that NFAT inhibitors in VIP^−/−^mice attenuate airway inflammation. However, hypertrophy in this context has not been studied.

VIP^−/−^ mice have biventricular dilated cardiomyopathy and primary pulmonary hypertension coincidental with strong overexpression of cardiac muscle genes, supporting the important role of VIP in maintaining homeostasis of the heart.
